# Sex distinctive patterns in the association between serum bicarbonate and uric acid levels among healthy adults. Qatar biobank data

**DOI:** 10.3389/fmed.2023.1021217

**Published:** 2023-06-02

**Authors:** Wisam Nabeel Ibrahim, Zumin Shi, Atiyeh M. Abdallah, Marawan Abdelhamid Abu-Madi

**Affiliations:** ^1^Department of Biomedical Sciences, College of Health Sciences, QU Health, Qatar University, Doha, Qatar; ^2^Department of Human Nutrition, College of Health Sciences, QU Health, Qatar University, Doha, Qatar

**Keywords:** bicarbonate, uric acid, gender, glomerular filtration rate, Qatar biobank

## Abstract

**Background:**

Uric acid is the final product of purine metabolism and is a potent plasma antioxidant but with pro-inflammatory effects. At high levels, it may increase the risk of developing multiple chronic diseases, such as gout, atherosclerosis, hypertension, and renal diseases. The aim of this study was to assess the sex-specific association between serum bicarbonate and uric acid levels among healthy adults.

**Methodology:**

This retrospective cross-sectional study included 2,989 healthy Qatari adults (36.4 ± 11.1  years) from the Qatar Biobank database. Serum uric acid and bicarbonate levels were estimated alongside other serological markers. Participants free from chronic diseases were divided into four quartiles based on serum bicarbonate levels. The sex-specific relationship between serum bicarbonate and uric acid levels was assessed through univariate and multivariate analyses.

**Results:**

In men, low serum uric acid levels were significantly associated with higher quartiles of serum bicarbonate levels after adjusting for age. The association remained significant after further adjustment for BMI, smoking, and renal function. The subgroup analysis using the restricted cubic spline method confirmed a significant dose–response association between the variation coefficients of uric acid by serum bicarbonate level in men with adjustments for age, BMI, smoking, and renal function. In women, no significant association was found between quartiles of serum bicarbonate and uric acid levels following the same adjustments. However, using the restricted cubic spline method, a significant bidirectional relation was demonstrated between serum bicarbonate and the variation coefficients of uric acid that were positive for serum bicarbonate levels below 25 mEq/L and negative at higher levels.

**Conclusion:**

Serum bicarbonate levels are linearly associated with reduced serum uric acid levels among healthy adult men, which may be a potential protective factor against hyperuricemia-related complications. Further research is needed to determine the underlying mechanisms.

## Introduction

Uric acid is a heterocyclic compound formed in the liver and intestines by the oxidation of hypoxanthine and xanthine via the xanthine oxidase enzyme, which is the end metabolic product of purine catabolism (1). In contrast to other mammals that possess uricase, which helps in achieving low serum uric acid levels, the normal level of uric acid in humans ranges from 155 to 357 μmol/L in women and up to 428 μmol/L in men (2). Uric acid is also a potent reducing agent and covers approximately 50% of the plasma antioxidant capacity (3, 4). However, hyperuricemia is a pro-inflammatory condition associated with high expression of inflammatory markers that can lead to several pathologies (5).

High levels of serum uric acid can precipitate easily as urate salt crystals in the kidneys and joints, leading to kidney stones, gout arthritis, and may independently increase the risk for several chronic diseases, such as coronary artery disease, hypertension, renal diseases, diabetes, and metabolic syndrome (3, 6).

Several population cohorts have confirmed the association of hyperuricemia with adverse outcomes, including renal and cardiovascular diseases. Hyperuricemia independently increases the risk of cardiovascular morbidity and mortality, impairs renal function by lowering the estimated glomerular filtration rate and increasing the urine albumin-to-creatinine ratio in patients with type 1 diabetes (7). Consistently, other epidemiological studies have confirmed the independent risk of hyperuricemia in developing chronic renal diseases, regardless of having diabetes mellitus diseases (8). Hyperuricemia may also increase the risk of ischemic and hemorrhagic strokes in a dose–response pattern, as evidenced by a meta-analytic study involving over 65,000 participants (9). Uric acid-lowering agents have also been shown to be beneficial in lowering the risk of cardiovascular events, such as coronary artery disease and heart failure (10).

The common risk factors for hyperuricemia include old age, male sex, alcohol use, obesity, renal diseases, and diabetes mellitus (11). Hyperuricemia may be due to increased production, reduced elimination, or both. The overproduction of urate can happen due to purine-rich diets, genetic metabolic defects that increase purine production, and rapid cell turnover in tumors; however, these causes are rarely responsible for hyperuricemia (12). Problems in renal handling are responsible for 90% of hyperuricemia cases because 70% of uric acid is filtered through the kidneys, and 90% is reabsorbed back into the blood (13). Therefore, hyperuricemia may occur due to decreased glomerular filtration, decreased tubular secretion in renal diseases, or increased tubular reabsorption.

The main problem is attributed to the precipitation of uric acid due to its low solubility limit, which is set at the level of 404 μmol/L, a level that is notably close to its normal limit in plasma. Additionally, the solubility of uric acid is affected by the pH level, reducing by six-folds at an acidic pH of 5.3 (14). The body employs several buffer systems in plasma to prevent the deposition of uric acid crystals in the joints and maintain a stable pH. One such buffer system is the equilibrium between urate ions and hydrogen ions (15). Urate ions, which are weak acids, can combine with hydrogen ions to form uric acid, which is less soluble than urate ions. This buffering system is particularly important in the distal tubule of the kidney, where the concentration of hydrogen ions is high (15). Additionally, the kidneys selectively excrete uric acid by secreting hydrogen ions or bicarbonate ions into the urine, which affects the urine pH and uric acid solubility, thus playing a crucial role in maintaining the balance between urate production and excretion and preventing hyperuricemia (16). The bicarbonate buffer system, another important buffer system in plasma, involves the equilibrium between CO_2_ and HCO3-. Carbonic anhydrase, an enzyme found in red blood cells, catalyzes the reversible reaction between carbon dioxide and water to form carbonic acid, which dissociates into hydrogen ions and bicarbonate ions. This buffer system plays a significant role in maintaining a stable pH in plasma and preventing the precipitation of uric acid (17).

Several reports have confirmed the therapeutic properties of serum bicarbonate in improving the prognosis of patients with chronic renal diseases and facilitating the renal handling of many electrolytes and drugs (18, 19). Bicarbonate is the endogenous buffer that helps to maintain the blood pH within its normal range, with a normal level of about 25 mEq/L. The protective effects of bicarbonate on renal function have been confirmed in several studies, in which its supplementation as sodium bicarbonate helped in reducing the mortality of chronic kidney diseases by improving the glomerular filtration (20). Therefore, the present study aimed to evaluate the association of serum bicarbonate levels with serum uric acid among the healthy Qatari population to delineate the possible confounding factors contributing to the association. By understanding the association between serum bicarbonate and uric acid levels, we can potentially identify new targets for the prevention and treatment of hyperuricemia-associated morbidities.

## Methodology

### Ethical statement

The study protocol was approved by the institutional review board of Qatar University and Qatar Biobank (E-2018-QBB-RES-ACC-0112-0054). Before the study commenced, written informed consent was obtained from all participants enrolled in the study. Blood samples were collected from Qatar Biobank, a research facility that collects and stores biological samples from the Qatari population for use in biomedical research. This facility operates under strict ethical guidelines to ensure the protection of participants’ rights and privacy.

### Subjects

The study enrolled a total of 2,989 healthy adult volunteers between the ages of 18 to 70 years old. To ensure the validity of the study results, exclusion criteria were applied to exclude participants with chronic disorders of the heart, lung, liver, kidney, and brain, as well as those with diabetes, high cholesterol, and high blood pressure. After obtaining written informed consent from the participants, blood samples were collected by a registered nurse at Qatar Biobank. To obtain socio-demographic characteristics, a self-administered questionnaire was designed to collect information on age, height, weight, body mass index (BMI), sex, and smoking history. Furthermore, during the interview session, the participants’ past medical history and other health-related information were also collected.

### Biochemical analysis

The blood samples were obtained from the participants following an overnight fasting period. A comprehensive panel of biochemical tests was performed to measure various serum analyses, including uric acid, calcium, phosphorus, and serum creatinine. Serum uric acid levels were measured using an enzymatic (uricase) colorimetric assay with absorbance measured at 520 nm using Roche Cobas 6,000 analyzer, following the standard protocol of chromogen incubation with serum sample for 30 min. The serum bicarbonate levels were measured using an enzymatic method (phosphoenolpyruvate carboxylase) with absorbance measured at 380/410 nm using Roche Cobas 6,000, following the standard procedure. Both tests were conducted in a laboratory setting with high precision and accuracy.

The estimated glomerular filtration rate (eGFR) is a measure of how well the kidneys are filtering waste from the blood. It is calculated using a formula that considers the level of serum creatinine, a waste product from muscles, as well as age, sex, and race. The equation used to estimate the GFR in this study was the Modification of Diet in Renal Disease (MDRD) equation (21), which is as follows:


eGFR=175×serum creatinine^−1.154×age^−0.203×0.742if female×1.212if African American.

In this equation, serum creatinine is measured in mg/dL, and age is measured in years. The constants 175, 0.742, and 1.212 are used to adjust for differences in race and sex. The MDRD equation is widely used in clinical practice as a way to estimate kidney function and is considered to be more accurate than using serum creatinine alone.

### Statistical analysis

The statistical analysis was performed using Stata 17 (Stata Statistical Software: Release 17. College Station, TX: Stata Corp LLC, United States). The data were presented as mean values with standard deviation (SD) or frequencies with percentages. Univariate analyses were conducted using appropriate statistical tests such as One-way ANOVA and Chi-square tests. A scatter plot with fractional polynomial fitted line was utilized to visually demonstrate the relationship between bicarbonate and uric acid levels in both men and women. The unadjusted association between serum bicarbonate and uric acid levels was evaluated by Pearson correlation analysis. Furthermore, a multiple regression model analysis was performed to assess the independent association between serum bicarbonate and uric acid levels after controlling for various confounding factors. Subgroup analyses were also conducted. To investigate the effect modification of age, obesity, and smoking on the relationship between serum bicarbonate and uric acid levels, multiplicative interactions between the quartiles of bicarbonate and these factors were assessed by adding a product term of the two variables in the multivariable model. The level of statistical significance was set at *p* < 0.05, and all statistical tests were two-tailed.

## Results

### Study characteristics

Table 1 displays the baseline characteristics of the study participants categorized by quartiles of serum bicarbonate levels. The study included 2,989 Qatari participants, with a mean age of 36.4 ± 11.1 years. The mean serum bicarbonate level was found to be within the normal range at 26.02 ± 2.12 mEq/L. Univariate analysis revealed a significant association between the quartiles of serum bicarbonate levels and various variables such as age, sex, body mass index (BMI), smoking status, renal function, and serum uric acid levels (value of *p* < 0.01).

**Table 1:** Sample characteristics per quartiles of bicarbonate among participants attending the Qatar Biobank Study.

	Total *N* = 2,989	Q1 *N* = 750	Q2 *N* = 918	Q3 *N* = 582	Q4 *N* = 739	value of *p*
Bicarbonate (mEq/L)	26.02 (2.12)	23.32 (1.05)	25.51 (0.47)	26.86 (0.30)	28.72 (1.07)	
Age	36.4 (11.1)	34.2 (10.4)	36.3 (11.0)	37.0 (11.3)	38.3 (11.4)	<0.001
Sex						<0.001
Men	1,436 (48.0%)	252 (33.6%)	408 (44.4%)	295 (50.7%)	481 (65.1%)	
Women	1,553 (52.0%)	498 (66.4%)	510 (55.6%)	287 (49.3%)	258 (34.9%)	
Smoking						<0.001
Non-smoker	2,027 (67.8%)	530 (70.7%)	649 (70.7%)	395 (67.9%)	453 (61.3%)	
Ex-smoker	189 (6.3%)	48 (6.4%)	47 (5.1%)	41 (7.0%)	53 (7.2%)	
Current smoker	773 (25.9%)	172 (22.9%)	222 (24.2%)	146 (25.1%)	233 (31.5%)	
Uric acid (umol/L)	296.53 (81.89)	283.29 (85.55)	296.08 (82.54)	297.58 (81.59)	309.69 (75.26)	<0.001
BMI (kg/m^2^)	28.4 (5.9)	28.8 (6.3)	28.8 (5.8)	28.1 (5.6)	27.8 (5.6)	0.002
Calcium (mmol/L)	2.28 (0.08)	2.27 (0.08)	2.28 (0.07)	2.28 (0.08)	2.29 (0.07)	<0.001
Phosphorus (mmol/L)	1.15 (0.17)	1.14 (0.16)	1.15 (0.16)	1.16 (0.18)	1.16 (0.17)	0.25
eGFR (mL/min/1.73 m^2^)	111.34 (15.07)	115.11 (14.87)	111.47 (14.83)	110.28 (14.80)	108.21 (14.96)	<0.001
eGFR < 90 mL/min/1.73 m^2^	257 (8.6%)	43 (5.7%)	77 (8.4%)	52 (8.9%)	85 (11.5%)	0.001

Continuous data were described as means and standard deviations (SD). BMI, body mass index; eGFR, estimated glomerular filtration rate – normal values are ≥ 90 mL/min/1.73 m^2^; The normal level of serum bicarbonate is 22–29 mEq/L, uric acid – male: 208–428 μmol/L: female: 155–357 μmol/L; Calcium – 2.2 to 2.7 mmol/L; phosphorus – 1.12 to 1.45 mmol/L.

Furthermore, the results indicate that serum uric acid levels increased gradually with the increase of serum bicarbonate levels (value of *p* < 0.001). Age, sex distribution, BMI, and the number of smokers also showed a gradual increase with the increase in bicarbonate levels. However, the estimated glomerular filtration rate showed a gradual decrease with the increase in serum bicarbonate levels (value of *p* < 0.001).

### Association between serum bicarbonate and uric acid levels

**Figure 1:**
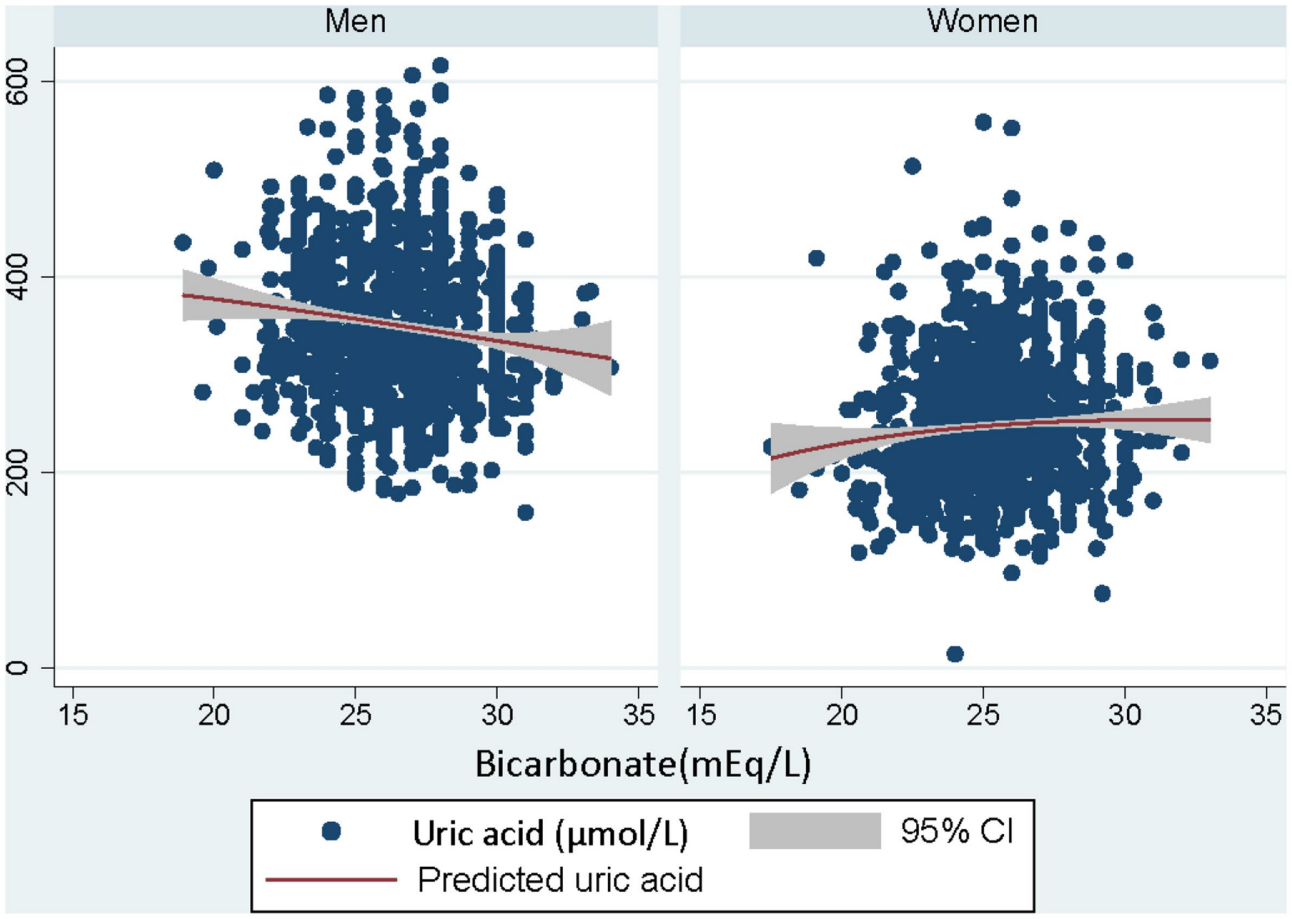
Illustration on the correlation analysis between serum bicarbonate and uric acid levels according to sex. Each dot in the scatter plot represents a study participant, and the trend lines indicate the overall correlation between the two variables for men and women separately.

The association between serum bicarbonate and uric acid levels was further explored with sex-specific correlation patterns. In men, a negative correlation between serum bicarbonate and uric acid levels was observed without any adjustments, as demonstrated in Figure 1. However, in women, no significant association was evident between serum bicarbonate and uric acid levels. These findings highlight the importance of considering sex-specific differences when exploring the relationship between serum bicarbonate and uric acid levels.

To analyze the association between serum bicarbonate and uric acid levels, a multivariate regression analysis with variably adjusted associations was conducted, as shown in Table 2. Two models were adjusted, and compared to the first quartile, the second, third, and fourth quartiles of serum bicarbonate were found to be independently associated with lower serum uric acid levels after adjustment for age, BMI, smoking, and kidney function.

**Table 2:** Regression coefficients (95%CI) for the association of serum uric acid by quartiles of bicarbonate in men and women Qatari adults attending Qatar Biobank.

	Serum Bicarbonate level							
	Q1	Q2		Q3		Q4		*p* value
*Men*								
Model 1	0.00	−4.05	(−24.76–−3.35)	−18.74	(−30.19–−7.29)	−25.79	(−36.17–−15.40)	0.000
Model 2	0.00	−12.32	(−22.69–−1.95)	−11.50	(−22.65–−0.35)	−15.59	(−25.88–−5.30)	0.011
*Women*								
Model 1	0.00	6.68	(−0.54–13.91)	−0.01	(−8.54–8.53)	1.34	(−7.70–10.38)	0.980
Model 2	0.00	6.48	(−0.49–13.45)	3.13	(−5.14–11.41)	3.51	(−5.19–12.21)	0.492

Model 1 adjusted for age. Model 2 further adjusted for BMI, smoking, and eGFR < 90 mL/min/1.73 m^2^.

To further investigate the relationship between serum bicarbonate and uric acid levels in different subgroups, Table 3 shows the results of the subgroup analyses. There was no significant interaction between quartiles of bicarbonate and age, obesity, and kidney function with uric acid in both men and women. However, a significant interaction was observed between bicarbonate and smoking in relation to uric acid in men.

**Table 3:** Subgroup analyses of the association between quartiles of bicarbonate and uric acid.

	Quartiles of bicarbonate					
	Q1	Q2	Q3	Q4	*p*	*p* for trend
*Men*						
Age						0.220
<40 years	0.00	5.56 (−2.30, 13.41)	−2.05 (−11.86, 7.76)	−3.06 (−14.36, 8.23)	0.528	
> = 40 years	0.00	8.30 (−5.43, 22.03)	10.92 (−4.08, 25.91)	9.32 (−5.22, 23.85)	0.221	
Smoking						0.021
Non-smoker	0.00	3.92 (−3.30, 11.14)	−0.27 (−8.86, 8.32)	1.80 (−7.25, 10.86)	0.893	
Ex-smoker	0.00	8.65 (−31.41, 48.70)	40.42 (−11.60, 92.44)	44.41 (−4.08, 92.89)	0.031	
Current smoker	0.00	50.85 (16.57, 85.13)	25.10 (−12.06, 62.27)	3.97 (−33.29, 41.23)	0.627	
eGFR < 90 mL/min/1.73 m^2^						0.143
No	0.00	5.08 (−1.94, 12.09)	2.62 (−5.73, 10.96)	4.38 (−4.40, 13.17)	0.386	
Yes	0.00	27.94 (−11.04, 66.92)	2.71 (−43.45, 48.87)	−7.87 (−53.16, 37.43)	0.566	
BMI > =30						0.538
No	0.00	9.88 (1.83, 17.92)	2.05 (−7.57, 11.68)	3.33 (−6.91, 13.56)	0.671	
Yes	0.00	1.39 (−11.51, 14.29)	3.61 (−11.50, 18.72)	3.25 (−12.37, 18.88)	0.620	
*Women*						
Age						0.334
<40 years	0.00	−18.81 (−32.31, −5.32)	−17.82 (−32.28, −3.36)	−21.49 (−34.81, −8.17)	0.010	
> = 40 years	0.00	−1.87 (−18.20, 14.45)	0.47 (−17.27, 18.21)	−6.19 (−22.53, 10.15)	0.473	
Smoking						0.672
Non-smoker	0.00	−4.54 (−21.12, 12.03)	−3.05 (−20.58, 14.49)	−13.60 (−29.81, 2.60)	0.079	
Ex-smoker	0.00	1.26 (−37.78, 40.30)	−10.18 (−48.67, 28.31)	−8.23 (−45.24, 28.78)	0.535	
Current smoker	0.00	−20.10 (−34.54, −5.67)	−17.74 (−33.77, −1.71)	−17.38 (−32.06, −2.70)	0.078	
eGFR < 90 mL/min/1.73 m^2^						0.321
No	0.00	−13.30 (−24.01, −2.60)	−10.53 (−22.08, 1.02)	−17.94 (−28.57, −7.31)	0.005	
Yes	0.00	−0.97 (−42.01, 40.06)	−13.50 (−56.67, 29.67)	8.70 (−32.43, 49.82)	0.565	
BMI > =30						0.113
No	0.00	−16.49 (−29.43, −3.56)	−18.52 (−32.05, −4.99)	−17.98 (−30.38, −5.59)	0.025	
Yes	0.00	−7.81 (−25.38, 9.77)	4.05 (−15.87, 23.96)	−12.52 (−31.53, 6.50)	0.399	

Models adjusted for age, BMI, smoking, and eGFR<90 mL/min/1.73 m^2^. Stratification variables were not adjusted in the corresponding models.

**Figure 2:**
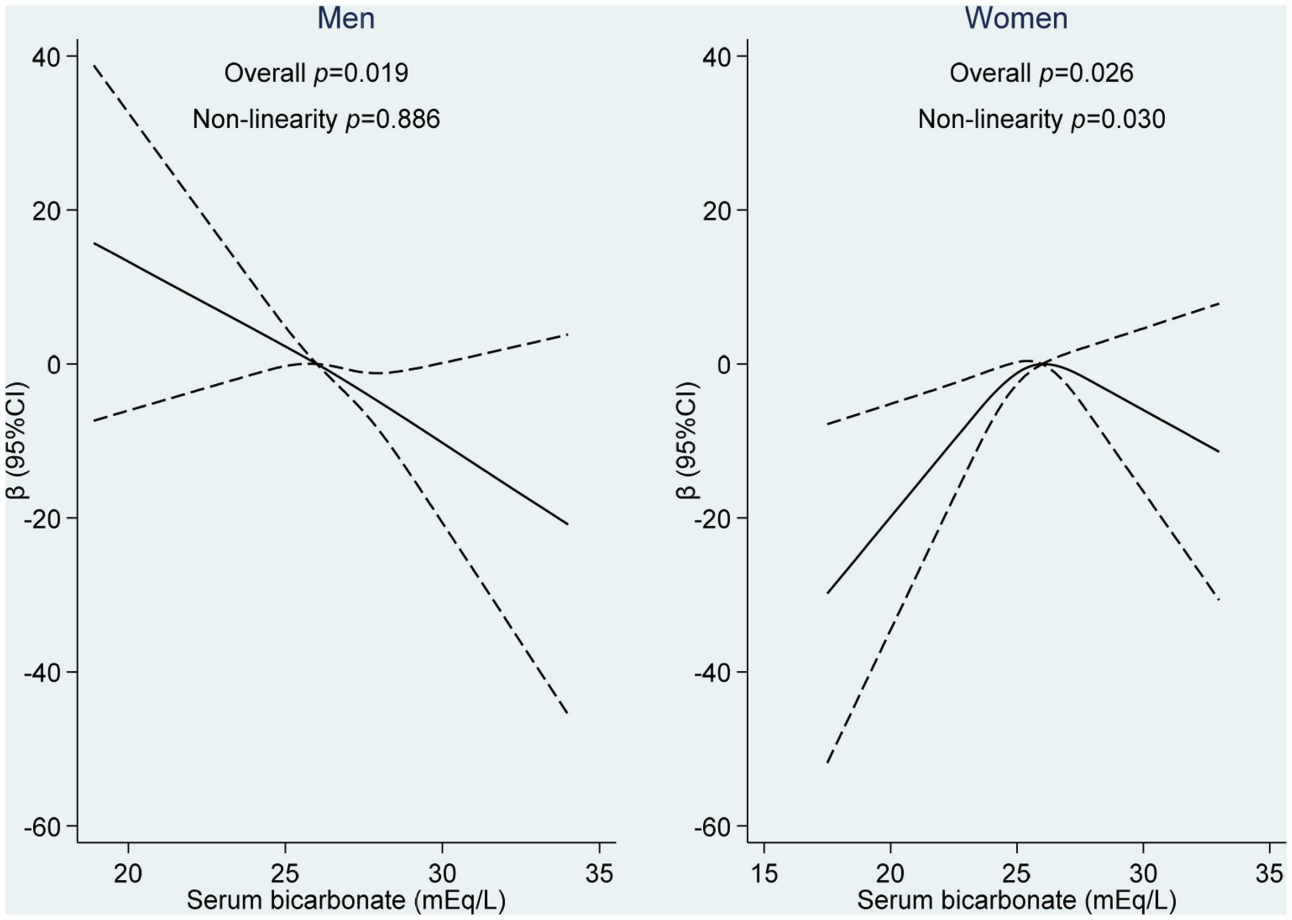
Non-linear association between serum bicarbonate and uric acid levels among adults attending Qatar Biobank. The restricted cubic spline regression model using three knots in males and females (10th, 50th, and 90th percentiles of serum bicarbonate) was used to examine the dose–response relationship between serum bicarbonate and uric acid levels, after adjusting for age, BMI, smoking, and eGFR.

To further investigate the dose–response association between serum bicarbonate and uric acid levels, a restricted cubic spline regression analysis was performed in men after adjusting for age, BMI, smoking, and renal function. The analysis revealed a significant linear association between serum bicarbonate and the regression coefficients of uric acid, indicating a dose–response effect (Figure 2). However, in women, no significant association was observed between quartiles of serum bicarbonate and uric acid levels after the same adjustments were made.

Interestingly, the same restricted cubic spline method was used to analyze the association between serum bicarbonate and the variation coefficients of uric acid in women. The results demonstrated a significant association with a binomial distribution at the serum bicarbonate level of 25 mEq/L. The association between serum bicarbonate and the variation coefficients of uric acid appeared to be positive at lower levels and negative at higher levels, as shown in Figure 2.

## Discussion

The results of this study showed an interesting specific pattern in the association between serum bicarbonate and uric acid levels among men and women. In men, the beta coefficient of serum uric acid was linearly and negatively associated with serum bicarbonate level. While among women, the association seemed to have a dimorphic pattern. Interestingly, the cut off value of serum bicarbonate level for this dimorphic pattern was 25 mEq/L. Serum bicarbonate values lower than this limit were in a positive linear association with serum uric acid level. This cutoff point might be attributed to the corresponding pH limit for acidosis in the blood and kidney tubules (22). This may consequently affect the level of uric acid, with lower pH values promoting the formation of uric acid crystals, while higher pH values facilitate uric acid dissolution.

Several reports confirmed the protective properties of bicarbonate in maintaining renal function (23, 24). Consistently, a U-shaped relationship has been identified between serum bicarbonate levels and the prevalence and outcomes of chronic kidney diseases, suggesting that low and high serum bicarbonate concentrations may be associated with dimorphic outcomes (25).

Thus, levels of bicarbonate lower than 25 mEq/L are associated with metabolic acidosis and bad prognosis in chronic renal disease patients. Furthermore, it has been observed that an acidic environment, characterized by a blood pH below the optimal range, increases the risk of hyperuricemia and its associated complications. Conversely, an alkaline environment can contribute to enhanced uric acid excretion by the kidneys.

In our study, the dimorphic association was only observed among women and regrettably, there is a lack of similar studies conducted on Middle Eastern or any other populations. Currently, there are no available reports to elucidate the sex-specific pattern of association between bicarbonate and uric acid. Thus, in an attempt to explore this uncharted territory, we have relied on indirect evidence, which suggests that sex hormones may play a role in renal function, diseases, and uric acid handling, in addition to the observed differences in renal function or structure between sexes.

Hyperuricemia, which is a condition characterized by high levels of uric acid in the blood, is primarily caused by the impaired reabsorption or reduced excretion of uric acid in the kidney (23). The regulation of uric acid levels in the proximal convoluted tubule of the nephron involves different transporter proteins such as URAT1, GLUT9, and ABCG2 (24, 25). Dysfunction of these transporters can lead to impaired urate handling and subsequently result in hyperuricemia and gout. Interestingly, there is a connection between the renal transporters of uric acid and the female sex hormone estrogen, which may explain the observed sex-specific differences in uric acid levels. Estrogen has been found to have a uricosuric effect by modulating the activity and expression of these transporters. Specifically, estrogen increases the expression of OAT1 and OAT3, promoting uric acid uptake into renal cells and facilitating its secretion into the tubular lumen. At the same time, it downregulates URAT1 expression, reducing uric acid reabsorption and further promoting its excretion (26). Moreover, estrogen can influence renal bicarbonate handling by upregulating the renal sodium-bicarbonate cotransporter (NBC), which enhances bicarbonate reabsorption, leading to a more alkaline environment that favors uric acid dissolution and excretion (27).

This observation is corroborated by the fact that men are four times more likely to develop hyperuricemia compared to women. However, after menopause, women appear to lose this protective advantage, as their risk of developing hyperuricemia increases fourfold (28, 29). Interestingly, these changes seem to diminish in postmenopausal women when hormone replacement therapy is initiated, suggesting that female sex hormones play a role in modulating the renal handling of uric acid. These combined effects of estrogen on uric acid transporters and renal bicarbonate handling may contribute to the observed sex-specific differences in uric acid levels and excretion.

In addition, other physiological differences in the renal function in the two sexes may contribute in this sex distinctive dimorphism (30–32). Women seem to have better renal function compared to males by having less risk to develop hypertension, chronic renal diseases, and better survival rates among patients with chronic renal diseases. However, this privilege is lost in the post-menopausal period or when renal pathologies are involved leading to more aggressive outcomes compared to men (33). Consequently, serum uric acid tends to increase dramatically in malfunctioning women’s kidneys leading to more hyperuricemia associated morbidities (34).

Thus, women with hyperuricemia are found to be at higher risk to develop hypertension compared to their male counterparts (35). As such, the risk of developing chronic renal diseases is more common in hyperuricemia women compared to hyperuricemia men.

Despite these findings, there is currently no clear understanding of the underlying mechanism behind the sex-specific differences in the association between bicarbonate and uric acid. Therefore, indirect evidence such as sex differences in renal function or structure and the effects of sex hormones on renal function, diseases, and uric acid handling may help in shedding light on this relationship. The study findings highlight the importance of understanding the molecular mechanisms underlying the sex differences for the development of targeted therapeutic strategies for managing uric acid-related disorders in both men and women.

Limitations of the current study includes the recruitment of healthy adults with basically normal bicarbonate levels. Including patients with abnormally high and low levels of bicarbonate would provide novel insights into the relation between the variables. Thus, a case control study design including patients with metabolic acidosis and alkalosis might improve the observations to the relation between bicarbonate and uric acid in both genders. Multiple measurements of serum bicarbonate level may help to eliminate the effect of the diurnal variation or other causes of physiological variation to improve the precision of findings. In this study, patients with respiratory, renal diseases and diabetes mellites were excluded to exclude extreme bicarbonate values and improve the research findings. In addition, the cross-sectional design may not help in proving a causal relationship between bicarbonate and serum uric acid levels. Thus, a longitudinal study design might improve the current observation with multiple measurements of bicarbonate level. The study mandates more experimental studies *in vitro* and *in vivo* to elucidate the molecular mechanisms in the relation to investigate the opportunities of possible therapeutic applications in hyperuricemia patients.

## Conclusion

The study confirmed sex distinct patterns in the relation between bicarbonate and uric acid levels in which serum bicarbonate level was negatively associated with serum uric acid among healthy Qatari men. While in women, bicarbonate ion was positively correlated with uric acid at sub physiological levels. Understanding the complex relationship between sex, serum bicarbonate, and uric acid levels is essential for the development of tailored therapeutic strategies for managing conditions such as gout, hyperuricemia, and metabolic acidosis. Future research should focus on elucidating the underlying mechanisms and sex-specific factors that contribute to these differences, as well as evaluating the potential benefits of personalized interventions based on sex, hormonal status, and acid–base balance.

## Data availability statement

The datasets presented in this article are not readily available because the data used and generated in this study are subject to restrictions and can only be requested from Qatar Biobank. Requests to access the datasets should be directed to https://www.qatarbiobank.org.qa.

## Author contributions

WI conceptualized the topic, wrote, and edited the manuscript, and contributed to data curation and statistical analysis. AMA edited the manuscript. ZS completed the statistical analysis and reviewed the manuscript. MA-M project administration and reviewing the manuscript. All authors contributed to the article and approved the submitted version.

## Conflict of interest

The authors declare that the research was conducted in the absence of any commercial or financial relationships that could be construed as a potential conflict of interest.

## Publisher’s note

All claims expressed in this article are solely those of the authors and do not necessarily represent those of their affiliated organizations, or those of the publisher, the editors and the reviewers. Any product that may be evaluated in this article, or claim that may be made by its manufacturer, is not guaranteed or endorsed by the publisher.
